# Radiographic closure time of appendicular growth plates in the Icelandic horse

**DOI:** 10.1186/1751-0147-49-19

**Published:** 2007-07-17

**Authors:** Eric Strand, Linn Camilla Braathen, Mia C Hellsten, Lisel Huse-Olsen, Sigridur Bjornsdottir

**Affiliations:** 1 Equine Teaching Hospital, Norwegian School of Veterinary Science, P.O.Box 8146 Dep. N-0033 Oslo, Norway; 2 Agricultural Authority of Iceland, Austurvegur 64, 800 Selfoss, Iceland

## Abstract

**Background:**

The Icelandic horse is a pristine breed of horse which has a pure gene pool established more than a thousand years ago, and is approximately the same size as living and extinct wild breeds of horses. This study was performed to compare the length of the skeletal growth period of the "primitive" Icelandic horse relative to that reported for large horse breeds developed over the recent centuries. This information would provide practical guidance to owners and veterinarians as to when the skeleton is mature enough to commence training, and would be potentially interesting to those scientists investigating the pathogenesis of osteochondrosis. Interestingly, osteochondrosis has not been documented in the Icelandic horse.

**Methods:**

The radiographic closure time of the appendicular growth plates was studied in 64 young Icelandic horses. The results were compared with previously published closure times reported for other, larger horse breeds. The radiographs were also examined for any signs of developmental orthopaedic diseases. In order to describe further the growth pattern of the Icelandic horse, the total serum alkaline phosphatase (ALP) activity was determined and the height at the withers was measured.

**Results:**

Most of the examined growth plates were fully closed at the age of approximately three years. The horses reached adult height at this age; however ALP activity was still mildly increased over baseline values. The growth plates in the digits were the first to close at 8.1 to 8.5 months of age, and those in the regions of the distal radius (27.4 to 32.0 months), tuber olecrani (31.5 to 32.2 months), and the stifle (27.0 to 40.1 months) were the last to close. No horse was found to have osteochondrosis type lesions in the neighbouring joints of the evaluated growth plates.

**Conclusion:**

The Icelandic horse appears to have similar radiographic closure times for most of the growth plates of its limbs as reported for large new breeds of horses developed during the past few centuries. It thus appears that different breeding goals and the intensity of breeding have not altered the length of the growth period in horses. Instead, it can be assumed that the pristine and relatively small Icelandic horse has a slower rate of growth. The appendicular skeleton of Icelandic horses has completed its bone growth in length at approximately 3 years of age, and therefore may be able to enter training at this time.

## Background

The growth plates at the distal radius and the tuber calcaneus have been used as indicators of skeletal maturity in Thoroughbred and Standardbred racing horses [1-3]. These breeds typically enter light training at 1.5 years of age, and formal race training at 2 years of age. It is widely thought among horsemen and veterinarians that Icelandic horses have open growth plates and grow in height until they are 4 to 5 years of age. As a result of this Icelandic horses do not receive demanding ridden training until they have reached that age. It is also thought that the slow growth rate over an extended period of time protects this breed from developing osteochondrosis, and other developmental orthopaedic disorders. To our knowledge, no study has been made regarding the closure time of the growth plates in the Icelandic horse, nor has anyone documented the existence of osteochondrosis in this breed.

The Icelandic horse has developed as an isolated breed since the settlement of the country in the 8th and 9th century. It originates from the medieval horse population of Norway and probably other countries in Scandinavia and the British Isles [4]. There is no evidence of introduction of new blood to the horse population since the end of the colonization period late in the 10th century [5]. The history of intense artificial selection of Icelandic horses is relatively short. Organized horse breeding based on different traits of conformation and performance under saddle has only been practised for one century [6-8]. For the last two decades, the breeding values have been obtained by a multiple-trait animal model (Best Linear Unbiased Prediction, BLUP) [9], accelerating the genetic improvement of the breed. The Icelandic horse is characterized by its ability to perform 4 or 5 gaits, and by its good health, and durability [10]. It is used for pleasure riding, long distance trekking and special gait competitions and has a wide distribution in Europe and North America [11].

The Icelandic horse is relatively small. Growth and development of the Icelandic horse was studied in the period 1970 – 1980 where the average height at the withers, measured by rod, was found to be 133 cm for five-year-old horses [12]. Measurements of horses presented for breeding evaluation in 2001 indicate an increase in the height of the breed in the last decades, as the average height of the mares was found to be 136.9 cm (128.0 – 146.0, SD 2.8) and for stallions 138.6 cm (130.0 – 151.0, SD: 3.0) [13].

The growth plate consists of a plate of hyaline cartilage, the physeal cartilage, and is seen on radiographs as a radiolucent line surrounded by diffuse relatively increased bone opacity. Endochondral ossification of the growth plates accounts for most of the linear growth of the long bones of the horse [14-16]. Cessation of this growth coincides with radiographic closure of the growth plate [15,16]. Radiographic closure has occurred when there is no radiolucent line visible in the physeal area. The closure time of selected growth plates of the limbs has been determined for some horse breeds [1-3,14,16-20].

Another method of evaluating the maturity of the skeleton is the measurement of biochemical parameters that are associated with growth and remodeling of bone tissue. Alkaline phosphatase (ALP) concentration in serum can be used to indicate the level of metabolic activity in the bone tissue of horses [21-23]. It reflects the active bone formation which accompanies skeletal modeling in the growing animal, and it decreases with age as the growth rate of the skeleton slows down [22-24].

The aims of this study were to determine the approximate radiographic closure time of the growth plates of the fore- and hind limbs of the pristine Icelandic horse, and to compare these closure times with those previously published for more recently developed large breeds of horses. The radiographs were also examined in order to document evidence of developmental orthopaedic disease such as osteochondrosis and osteochondral bone cysts in the neighbouring joints. In order to further describe the growth pattern of the Icelandic horse, the total serum alkaline phosphatase (ALP) activity was determined, and the height at the withers was measured. This information would provide practical guidance to owners and veterinarians as to when the skeleton is mature enough to commence formal ridden training, and would be potentially interesting to those scientists investigating the pathogenesis of osteochondrosis.

## Methods

### Horses

The material consisted of 64 Icelandic horses, including 38 mares, 15 stallions and 11 geldings. Thirty-eight of the horses were examined in Iceland in late September 2004 and 26 in Norway in January and April 2005. The age of the horses ranged from 47 days to 52 months at the time of examination. All the horses were born during the spring and summer months, the majority in May and June of each year. Each horse was examined one time. All the horses were found to be in good nutritional condition. Further information about the management was collected from the owners: In Iceland, most of the young horses that were not in training were kept out-of-doors on large pastures all year round. The foals were weaned at approximately 6 – 9 months of age, and were either stabled for the winter months or kept at pasture with access to open shelters. The feeding consisted of grazing from June to October/December, and haylage/silage ad lib out-of-doors during the winter. Mineral supplements were usually provided by salt licks. In this material, the three-and-a-half-year-old horses (42 – 46 months) were being saddle broken, and the four and-a-half-year-olds (48 – 52 months) were in light training at the time of examination. The management regimes in Norway were similar, except for supplementary feeding of grain to all horses from weaning, and that most of the horses in Norway were stabled during the winter months. The horses were privately owned, and intended for pleasure riding and gait competitions. They had no history of illness or injury.

### Radiographic examination

The horses were sedated with a combination of detomidine (Domosedan^®^, Orion Corporation, Turku, Finland) 10–40 μg/kg bwt and butorfanol (Torbugesic^®^, Fort Dodge Animal Health, Overland Park, Kansas, USA) 20–30 μg/kg bwt intravenously. Radiographs were taken using a 80 kV, 15 mA, 1.99 sec portable X-ray machine (Gierth HF 80/15 plus ULTRA LEICHT, Gierth X-Ray International GmbH, Riesa, Germany). The focal-film distance (FFD) was 100 cm, and regular speed screens were used.

The anatomical regions included in this study were: the phalanges for all horses 46 days to 24 months of age; and the carpus, elbow, hock, and stifle for all horses 8 months to 40 months of age. Two views, in the frontal (cranial to caudal) and sagittal (lateral to medial) plane, were taken for each region of the left thoracic and left pelvic limb. Sixteen different growth plates of the appendicular skeleton were examined. The radiographs were all interpreted by a panel consisting of at least three of the five authors and the classification of each physis was agreed upon by consensus.

**Figure 1:**
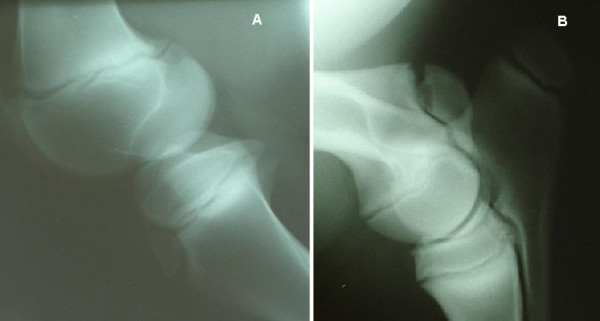
**Examples of fully open growth plates**. A) The proximal tibia, tuberositas tibia and distal femur of a 46-day-old foal. B) The tuber olecrani, proximal radius and distal humerus of a 4-month-old foal

**Figure 2:**
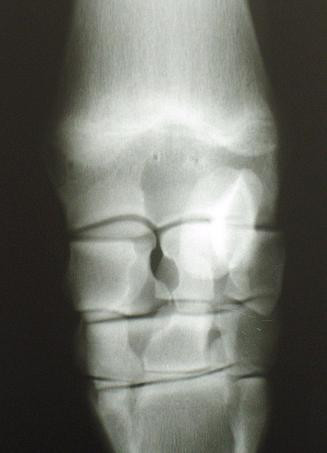
**Example of a growth plate classified as closing**. The carpus of a horse aged 26.7 months. The growth plate at the distal radius is classified as closing. Note the intermittently present radiolucent line surrounded by diffuse opacity (arrow).

**Figure 3:**
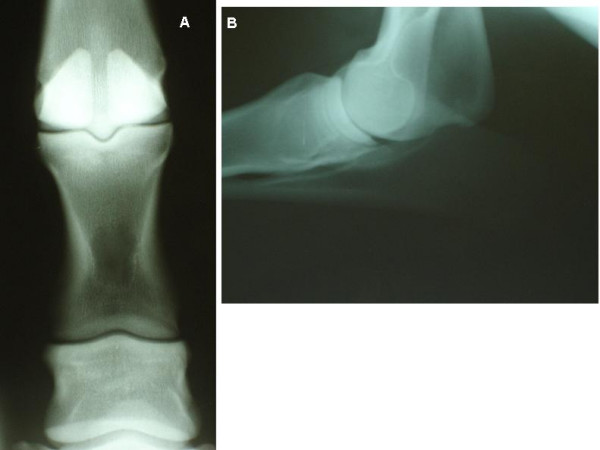
**Examples of fully closed growth plates**. A) Horse aged 22.8 months. Fully closed growth plates at the proximal second phalanx, proximal first phalanx and distal metacarpus. B) Horse aged 50.8 months. Fully closed growth plates at the proximal radius and distal humerus. Note the absence of any radiolucency and diffuse opacity in the region of the previous growth plate.

For the purpose of this study, the growth plates were classified as fully open, closing and fully closed, in order of advancing fusion of the growth plate [2,18,25,26]. A growth plate was classified as fully open when a distinctly radiolucent line could be observed spanning the whole extent of the growth plate region (Fig. 1). A growth plate was classified as closing when a radiolucent line was present in the growth plate area, but only intermittently and surrounded by diffuse relatively increased bone opacity (Fig. 2). A growth plate was classified as fully closed with total absence of the radiolucent line in the region of the previous growth plate in the two radiographic projections (Fig. 3). When there was a difference in appearance of the growth plates on separate views of the same area, the growth plate was classified according to the view that showed the lowest degree of fusion. Other subjective features of the growth plates such as width were noted. Time of closure for each growth plate was defined as the age range from the youngest horse observed with a fully closed growth plate, to the age after which all further horses examined had a fully closed growth plate.

### Alkaline phosphatase and height at the withers

Prior to sedation, blood was drawn from the external jugular vein into two 10 ml vials without supplement. The whole blood was centrifuged later the same day and the serum frozen to -18°C for later analysis or was analyzed directly. Total serum alkaline phosphatase (U/L) was measured with the Modified IFCC method [27]. The height at the highest point of the withers was measured with the horse standing square on a level surface. Most of the horses were measured under mild sedation because they were not used to extensive handling. In order to have reference values of total ALP of adult Icelandic horses, intravenous blood samples were collected from a control group which consisted of 11 reportedly healthy Icelandic horses at the age of 7 to 16 years.

## Results

### Radiographic examination

The growth plates in the first and second distal phalanges and the proximal third phalanx as well as the proximal Mt3 and Mc3 were all fully closed in the youngest horses in this study. The time of closure of the sixteen other growth plates examined are listed in Table 1, and in Table 2 these same results are listed together with published data from other horse breeds. The growth plates of the Icelandic horses were subjectively characterized as narrow in most of the regions studied, relative to those present in large horse breeds, although the width was not objectively measured.

**Table 1:** Radiographic closure time (age range in months) of appendicular growth plates in 64 Icelandic horses

**Growth plate**	**n**	**Growth plate fully open**	**Growth plate fully closed = Closure time**
Proximal second phalanx (TL)	35	1.5 – 6.1	8.1
Proximal first phalanx (TL)	37	1.5 – 4.3	8.1 – 8.5
Proximal second phalanx (PL)	35	1.5 – 4.3	8.1
Proximal first phalanx (PL)	37	1.5 – 6.1	8.1 – 8.5
Distal third metacarpal	37	1.5 – 4.3	8.1 – 8.5 (16.4)
Distal third metatarsal	37	1.5 – 6.1	8.1 – 14.9 (16.4)
Distal radius	55	1.5 – 22.9	27.4 – 32.0 (39.1)
Proximal radius	56	1.5 – 11.0	14.9
Tuber olecrani	55	1.5 – 26.7	31.5 – 32.2
Medial epicondyle of humerus	56	1.5 – 14.9	15.2
Distal humerus	56	1.5 – 4.3	8.8 – 11.0
Tuber calcanei	56	1.5 – 11.0	19.0 – 26.7
Distal tibia	56	1.5 – 11.0	15.3 – 19.0
Tuberositas tibiae	52	1.5 – 38.6	38.6 – 40.1
Proximal tibia	56	1.5 – 22.8	23.0 – 32.2 (38.6)
Distal femur	53	1.5 – 16.4	19.0 – 27.0

**Fully open **= distinct radiolucent line spanning the entire extent of the physis; **fully closed **= no radiolucency in the region of the physis. The **Radiographic closure time **was defined as the age range from the youngest horse observed with a fully closed physis, to the age after which all further horses had a fully closed physis. Single outliers which were in the "closing stage" are in parenthesis.

**Table 2:** Previously published reports of closure times (months) of the appendicular growth plates in different horse breeds together with the results for Icelandic horses in this study.

		**Growth plate**								
***Breed***	**n**	**Proximal second phalanx**	**Proximal first phalanx**	**Distal third metacarpal**	**Distal third metatarsal**	**Distal radius**	**Proximal radius**	**Tuber olecrani**	**Distal humerus**	**Tuber calcanei**

Brazilian Thorougbred [27]	20					20.9–27.6				
Thoroughbred [19]	800		8.0–14.0	8.0–14.0						
Thoroughbred [20]	53									16.0–24.0
American Standardbred [16]	113					26.0–35.0				
Standardbred [13]	14					24.2–31.9				
American and Italian Standardbred [15]	140					26.0–29.0				23.0–27.0
Arabian [14]	2	7.5–7.9	7.5–8.8	7.0–7.5	7.0–7.5	23.2–23.7	13.6–14.0	26.6–29.7	13.6–14.9	
Quarter Horse [12]	6						Ca. 18			
Thoroughbred-Quarter Horse Cross [11]	9		6.0–10.0 (tl)* 8.0–11.0 (pl)*	7.0–9.5 (18)**	9.0–12.5	24.0–25.5				
Brazilian Manga-larga [17]	7					24.6				
Finnhorse [18]	15					24.0–30.0				

Icelandic horse, current study	35–56**	8.1	8.1–8.5	8.1–8.5 (16.4)***	8.1–14.9 (16.4)***	27.4–32.0 (39.1)***	14.9	31.5–32.2	8.8–11.0	19.0–26.7

*tl = thoracic limb, pl = pelvic limb, **Depending on region, *** The closure times with single outliers are included in parentheses

### Alkaline phosphatase and height at the withers

**Figure 4:**
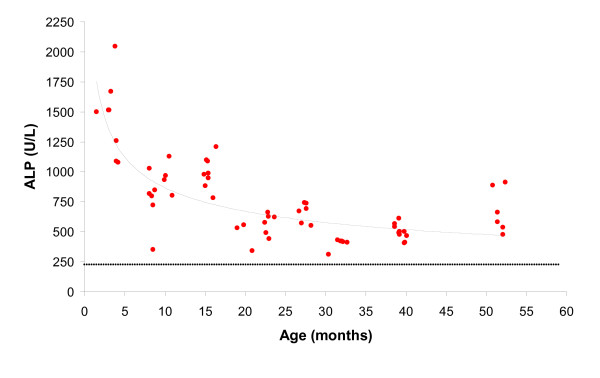
The relationship between alkaline phosphatase (ALP, in U/L) and age (in months) of 64 Icelandic horses with a fitted geometric curve. The dotted line at the ALP-level of 242.5 U/L represents the mean ALP value of the control group of 11 horses, which were 7 to 16 years of age.

**Figure 5:**
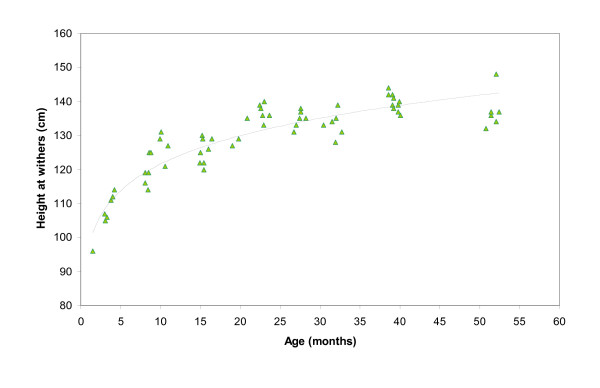
The relationship between height at the withers and age of 63 Icelandic horses. The fitted curve is a geometric model.

The results of the measurements of total ALP as well as the height at the withers are plotted against the age of the horses in Figures 4 and 5 respectively. A geometric trend line was added to the graphs in both figures. For comparison, the mean value of 242.5 U/L of total ALP of the control group of 11 adult horses was added to Figure 4 as a dotted horizontal line.

No signs of osteochondrosis or other developmental orthopaedic disease was found in the neighbouring joints of the evaluated growth plates.

## Discussion

A complete overview of the closure times of the appendicular growth plates requires either following a group of growing horses for several years, or studying a representative cross section of individuals at critical points of the development. Here, a cross-sectional design was chosen, as groups of individuals of the appropriate age could be captured on a few occasions during a calendar year. The material was haphazardly selected from farms in different locations in Iceland and Norway and is considered to be representative for the breed without known biases.

The closure times of the appendicular growth plates in the Icelandic horse listed in Table 1 were found to be similar to existing data for other horse breeds (see Table 2). The only exception was a tendency for the growth plates of the distal radius to close later in Icelandic horses, compared with Thoroughbred/Quarter horse crosses [14], Brazilian Thoroughbreds [26], Brazilian Mangalarga [18] and a limited material of Arabian horses [17]. However, few complete studies are presently available for comparison of our results. Many of the studies listed in Table 2 are based on a very limited number of young horses, and/or a limited number of growth plate regions. No previously published data could be found for many of the regions now investigated in the Icelandic horse. In general, the differences in the closure times of the growth plates appear to be minimal between breeds despite of the great variation in adult sizes. This suggests a slower growth rate in smaller breeds, such as the Icelandic horse. The consistent "subjective"observation of relatively narrow growth plates in this study, compared to the much wider growth plates observed in adolescent horses of large breeds, also supports this suggestion. Measuring the actual growth rate of the Icelandic horse would, however, have required measurements of the size at birth and was beyond the scope of this study.

The radiographic determination of growth plate closure is a result of subjective evaluation, and correct interpretation depends on many factors. To reveal the radiolucent cartilage at the growth plate, the x-ray beam must be aimed directly perpendicularly to the growth plate; otherwise overlapping bone tissue can be misinterpreted as evidence of fusion of the growth plate. Since the growth plates in many sites are not flat discs, but undulate to a variable degree, often in two or more directions, the problem of overlapping is often present also in good-quality radiographs [28]. In addition, the physeal cartilage becomes narrower with increasing age [29], which makes it more difficult to discern between fully open and partially fused growth plates. Therefore, to minimize interpretation difficulties, two views (cranio – caudal and lateral – medial) of each region were used. In some cases, it was still difficult to distinguish between "late" closing and fully closed. This is a possible explanation to the outliers in the present study. Other authors have also found what seem to be single outliers in their material [14,25].

Total serum alkaline phosphatase (total ALP) consists of fractions of several tissue-specific isoenzymes. In healthy young horses only two different isoenzyme fractions appear in the serum: liver and bone ALP [21]. The level of total ALP decreases with age, particularly during the first year of life, mainly due to the decrease of the bone fraction as the skeleton growth rate slows down with age [22,23]. In horses younger than one year, the bone fraction is 60% of the total ALP, while in horses over five years of age it has decreased to 20 % [22]. The same pattern in the changes of total ALP with age was observed in the Icelandic horse (see Fig 4). The plots followed a fitted geometric curve that was steepest in the first year of life, and had almost reached a horizontal line at 38 to 40 months. At this age the growth plates studied were all closed and the height at the withers seemed to have reached the adult level. However, the total ALP in the three- and four-year-old horses had still not decreased to the baseline value of the control group of 11 adult horses, and was also higher than in adult Icelandic horses in a previous study [30]. The mean total ALP in the four-year-old horses was 675.7 U/L, which was actually higher than in the three-year-olds that had a mean ALP of 497.4 U/L. Although all the growth plates in the current study were closed at the age of four years, it has been described that more proximal growth plates, for example in the pelvis and the vertebral column, can still be open at this age [31]. It is also known that considerable remodeling occurs at the physeal sites for a long time after radiological closure [16]. The four-year-old horses were in light training which has been reported to cause an increase in both liver and bone ALP in Swedish Standardbred trotters up to the age of three years [21].

Radiographic signs of developmental orthopaedic disease were not identified in this material nor in an earlier report based on radiographic examination of the tarsi of 614 Icelandic horses in the age of 6 – 12 years [32]. Thus no radiographic survey of Icelandic horses to date has demonstrated the existence of osteochondrosis type fragments.

## Conclusion

This study provides practical information for trainers and veterinarians working with the Icelandic horse. Traditionally, demanding ridden training of Icelandic horses commences at the age of 4 years at the earliest. According to the current study, the appendicular skeleton should be ready for increased load at 3 years of age, as most appendicular growth plates are closed by then. The results also suggest that the Icelandic horse, with its gene pool established over 1000 years ago, has approximately the same growth period as breeds of horses which have been especially selected for size during the past few centuries. In our study the Icelandic horse was also subjectively evaluated to have relatively narrow growth plates, relative to large horse breeds, in all age groups suggesting a slower growth rate. The growth rate of the Icelandic horse needs to be investigated further, as well as the association between growth rate and developmental orthopaedic abnormalities.

## Competing interests

The author(s) declare that they have no competing interests.

## Authors' contributions

The authors contributed equally to this work. All authors read and approved the final manuscript.
